# Proteomic Analysis of Kidney in Rats Chronically Exposed to Monosodium Glutamate

**DOI:** 10.1371/journal.pone.0116233

**Published:** 2014-12-31

**Authors:** Amod Sharma, Chaisiri Wongkham, Vitoon Prasongwattana, Piyanard Boonnate, Raynoo Thanan, Sirirat Reungjui, Ubon Cha’on

**Affiliations:** 1 Department of Biochemistry, Faculty of Medicine, Khon Kaen University, Khon Kaen, Thailand; 2 Department of Internal Medicine, Faculty of Medicine, Khon Kaen University, Khon Kaen, Thailand; Emory University, United States of America

## Abstract

**Background:**

Chronic monosodium glutamate (MSG) intake causes kidney dysfunction and renal oxidative stress in the animal model. To gain insight into the renal changes induced by MSG, proteomic analysis of the kidneys was performed.

**Methods:**

Six week old male Wistar rats were given drinking water with or without MSG (2 mg/g body weight, n = 10 per group) for 9 months. Kidneys were removed, frozen, and stored at –75°C. After protein extraction, 2-D gel electrophoresis was performed and renal proteome profiles were examined with Colloidal Coomassie Brilliant Blue staining. Statistically significant protein spots (ANOVA, *p*<0.05) with 1.2-fold difference were excised and analyzed by LC-MS. Proteomic data were confirmed by immunohistochemistry and Western blot analyses.

**Results:**

The differential image analysis showed 157 changed spots, of which 71 spots were higher and 86 spots were lower in the MSG-treated group compared with those in the control group. Eight statistically significant and differentially expressed proteins were identified: glutathione S-transferase class-pi, heat shock cognate 71 kDa, phosphoserine phosphatase, phosphoglycerate kinase, cytosolic glycerol-3-phosphate dehydrogenase, 2-amino-3-carboxymuconate-6-semialdehyde decarboxylase, α-ketoglutarate dehydrogenase and succinyl-CoA ligase.

**Conclusion:**

The identified proteins are mainly related to oxidative stress and metabolism. They provide a valuable clue to explore the mechanism of renal handling and toxicity on chronic MSG intake.

## Introduction

Monosodium glutamate (MSG) is a common food additive popular for its *umami* taste [1]. It is supplemented to processed foods and sprinkled onto foods, mostly in Asian cuisine. Although MSG is considered safe for the general population, chronic oral MSG intake [2] or injection [3] alters renal antioxidant systems and markers including lipid peroxidation byproducts in rats. Chronic MSG administration-induced oxidative stress was also seen in the liver and brain of rat [4], [5]. Moreover, long term consumption of MSG has been shown to enhance tubulo-interstitial fibrosis in rat kidneys [6], possibly due to oxidative stress.

Published data indicate that chronic MSG not only causes oxidative stress, kidney dysfunction [2] and kidney stone [6] but also distorts cytoarchitecture, increases glomerular hypercellularity and infiltration of inflammatory cells in the renal cortex [7]. The formation of reactive oxygen species (ROS) in kidney exposed to MSG overintake is considered a major contributor to their nephrotoxic effects leading to the cellular and functional damage [3]. However, the mechanisms governing key proteins behind MSG induced renal toxicity and renal handling of MSG overintake remains unexplored to date.

Proteomic approaches have been used for the better understanding on the underlying mechanisms of nephron-toxicity of various chemicals such as gentamicin, cisplatin, perfluorododecanoic acid [8], [9]. Therefore, the aim of the present study is to assess the effects of chronic MSG intake on patterns of renal protein expression in rats.

## Materials and Methods

### Animals and MSG treatment

MSG (99%-pure food-grade package) dissolved in drinking water was administered *ad libitum* to Wistar male rats to achieve a daily dose of 2 mg/g body weight as estimated by daily water intake measurements. Rats, 6-weeks-old (150–200 g), were allowed to acclimatise for 1 week (wk) and then randomly assigned to treatment or control groups, with 10 rats each in each group. They were kept at 25±2°C and 60% humidity with a 12-h light/dark cycle, and were housed 2–3 per cage on wood chips and provided a standard rat chow pellet (Perfect Companion Group, Thailand). All protocols complied with the guidelines of the Northeast Laboratory Animal Center (NELAC), Khon Kaen University, Thailand, and were approved by the Animal Ethics Committee of Khon Kaen University, Thailand.

### Preparation of kidney for immunohistochemistry and kidney proteins for 2-D gel electrophoresis

Rats were euthanized by intraperitoneal Nembutal injection after 9 months of MSG treatment. Left kidneys were removed and washed with cold normal saline, dissected, and fixed in 4% paraformaldehyde solution for histopathological analysis. Right kidneys were also removed and washed with cold normal saline, flash-frozen in liquid nitrogen, and stored at –70°C. Approximately 50 mg of renal tissue was minced on ice and homogenized with a hand-held tissue homogenizer in 50 µl of lysis buffer (7M urea, 2M thiourea, 4% CHAPS) containing the protease inhibitor cocktail (Roche Diagnostics). After a 1 h incubation at room temperature with occasional shaking, the homogenate was centrifuged at 30,000×g for 30 min at 4°C and the supernatant was collected. Protein concentrations of the samples were assayed using the Bradford method. Renal homogenate of the rats in each group were pooled.

### 2-D gel electrophoresis

A fixed amount of 150 µg of kidney protein from the pooled sample of both groups was mixed in thiourea rehydration solution (7M urea, 2M thiourea, 2% CHAPS, 60 mM DTT, 0.5% (v/v) IPG buffer pH 3–11, trace of bromophenol blue) to a volume of 125 µl which was then loaded onto 7 cm IPG Strip (pH 3–11 NL). Rehydration was performed using the IPGphor IEF system (50 µA for 12 h at 20°C). The first dimension IEF was performed at 20°C with the following parameters: 200 Vh, 303 Vh, 7,500 Vh and 3,000 Vh for a total 11,003 Vh according to the manufacturer’s protocol (GE Healthcare, Sweden). The strips were first equilibrated for 30 min in equilibration solution (pH 8.8) containing 75 mM Tris-HCl, 6M urea, 30% (w/w) glycerol, 2% (w/w) SDS and 1% DTT, then for an additional 30 min in equilibration solution (pH 8.8) containing 75 mM Tris-HCl, 6M urea, 30% (w/w) glycerol, 2% SDS and 2.5% iodoacetamide. The second dimension electrophoresis was carried out on the electrophoresis apparatus with 12% vertical SDS-PAGE slab gels (100 mm × 100 mm × 1 mm), run at a constant voltage of 100 V on ice for cooling until bromophenol blue reached the gel bottom. Individual gels of the two groups were run in pairs on 2-D gel electrophoresis. The electrophoresis of the each sample was repeated three times under the same condition.

### Visualization and image analysis of 2-D gel electrophoresis

A total of six gels were stained with Coomassie Brilliant Blue R 250 for visualization of spots. Briefly, the gels were incubated in the staining solution (0.2% Coomassie Brilliant Blue R 250 in 1∶1 ethanol and 20% trichloroacetic acid) with mild shaking for 2 h at room temperature, followed by destaining (8% acetic acid and 25% ethanol) for 2 h to clear background stain. The stained gels were scanned with 300 dpi resolution using ImageScanner III Lab Scan software (GE Healthcare, Sweden). Three pairs of gel images from the two groups were analyzed with ImageMaster 2D platinum 7 software (GE Healthcare, Sweden) for three times. Spot detection and spot matching were performed automatically and manually, respectively. The cut-off value was set at 1.2 fold increase or decrease. Statistical analysis was performed using ANOVA, and values of p<0.05 were considered statistically significant. Only proteins with significantly altered levels were excised for the identification using LC-MS.

### Peptide mass mapping using LC- MS and database search

The protein spots of interest were excised from the gel by the Ettan spot picker and washed twice with 20 mM Ambic, 20 mM Ambic/ACN (1∶1) and ACN for 10 min each. The gels were then treated with 10 mM DDT in 20 mM Ambic for 45 min at 56°C followed by 55 mM iodoacetamide in 20 mM Ambic for 30 min at room temperature. Next, washing steps involved two washes with 20 mM Ambic/ACN (1∶1) and one wash with ACN for 10 min each. For in-gel digestion, trypsin (Promega, USA) solution (20 ng/µl in 20 mM Ambic) was added and digested at 4°C for 30 min. Gel pieces were incubated with 25 mM Ambic overnight at 37°C. Peptides were extracted with 50% ACN/1% formic acid and dried using a centriVap centrifugal vacuum concentrator (Labconco Corporation, USA) for 2 h, followed by reconstruction with buffer (2% ACN/0.1% formic acid) and analysis with an ion trap mass spectrometer (Amazon speed ETD, Bruker, USA). The peptides obtained from the proteolytic digest were separated on the EASY column (10 cm, ID 75 µm, 3 µm, C18-A2; Thermo Scientific, USA) and eluted with a solution A (0.1% formic acid) to 35% solution B (0.1% formic acid in acetonitrile) for 25 min.

The eluted peptides were directly analysed by electrospray ionization. The instrument was under the service of Khon Kaen University Research Instrument Center, Thailand. The protein database search program MASCOT (http://www.matrixscience.com) was used to compare the peptide signature of the tryptic fragments with NCBI database restricted to taxonomies *Rattus norvegicus* (rat). For modification of peptides, cysteine carbamido-methylation and methionine oxidation were considered. The peptide tolerance and fragment mass tolerance were set to 0.5 Da. When 2 or more proteins with high scores were identified in the same spot, they were excluded from the analysis.

### Immunohistochemistry analysis

Routinely processed paraffin-embedded tissue blocks were sliced at 4 µm thickness. Tissue sections were deparaffinized, rehydrated, and antigen retrieval by boiling method. Increased permeability with 0.1% Triton-x 100/PBS then blocked endogenous peroxidase and non-specific binding by 0.3% H_2_O_2_ in methanol and 3% bovine serum albumin (BSA), respectively. Sections were then incubated overnight at room temperature with goat monoclonal anti-α-ketoglutarate dehydrogenase (anti-α-KGDH; dilution 1∶50, Santa Cruz Biotechnology, Santa Cruz, CA, USA) and anti-glutathione S-transferase P (anti-GSTP; dilution 1∶50, Santa Cruz Biotechnology, Santa Cruz, CA, USA). The sections were washed with phosphate-buffered saline (PBS, pH 7.4), incubated for 1 h at room temperature with rabbit anti-goat IgG peroxidase antibody (dilution 1∶250, DAKO Corporation, Hamburg, Germany), washed with phosphate-buffered saline (PBS, pH 7.4), and stained with 3,3′-diaminobenzidine tetrahydrochloride (DAB) (Sigma-Aldrich, USA).

### Western blot analysis

The protein was extracted from kidney tissues by extraction buffer (7M urea, 2M Thiourea, 4% w/v CHAPS and protease inhibitors). The protein concentrations were determined by using the Bradford method (Bio-Rad protein assay kit, CA, USA). Total protein extracts were separated by 12% SDS-PAGE electrophoresis. After electrophoresis, the proteins were electro-transferred to polyvinylidene difluoride (PVDF) membrane (GE Healthcare). The PVDF membrane was blocked with 3% BSA in phosphate-buffered saline-0.3% Tween20 (PBST), then were probed with goat monoclonal antibodies specific for α-KGDH, GSTP (dilution 1∶200, Santa Cruz Biotechnology, Santa Cruz, CA, USA) and mouse monoclonal β-actin (dilution 1∶200,000, Sigma-Aldrich, USA). After overnight incubation, the membranes were washed with PBST and incubated with HRP-conjugated secondary antibodies, rabbit anti-goat (dilution 1∶10,000, DAKO Corporation, Hamburg, Germany) and goat anti-mouse (dilution 1∶10,000, Invitrogen, Carlsbad, CA). The blots were visualized by SuperSignal enhanced chemiluminiscence (ECL) detection system (GE Healthcare) according to the manufacturer’s instructions. Data were analyzed using Student’s *t*-test. P values <0.05 were considered statistically significant.

## Results

**Figure 1:**
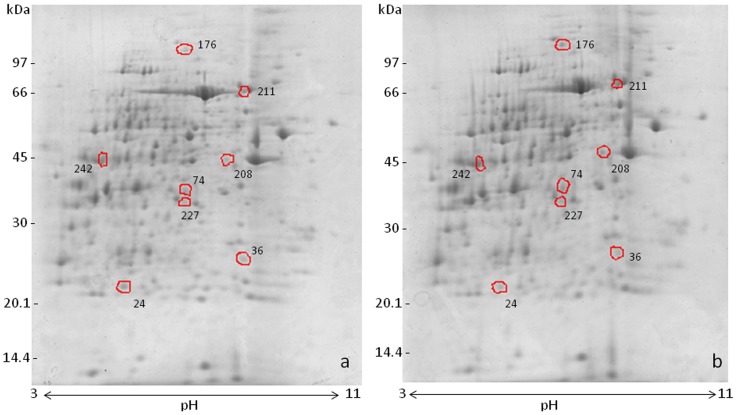
Two-dimensional gel electrophoresis of rat kidney lysate; Coomassie blue-stained gels from control (a) and MSG-treated rats (b). Proteins were resolved on 7 cm pH 3–11 IEF strips (NL) followed by SDS-PAGE (12%). The differentially expressed spots detected by the image Master 2D Platinum 7.0 software are circled. The gels shown are representative of three independent experiments.

A total of 252 spots matched between the MSG-treated and the control groups on the 2-D gel electrophoresis images. The differential image analysis showed 157 changed spots, of which 71 spots were higher and 86 spots were lower in the MSG-treated group compared with those in the control group. Eight statistically significant spots with >1.2 fold increase or decrease were processed for identification (**Fig. 1**).

**Figure 2:**
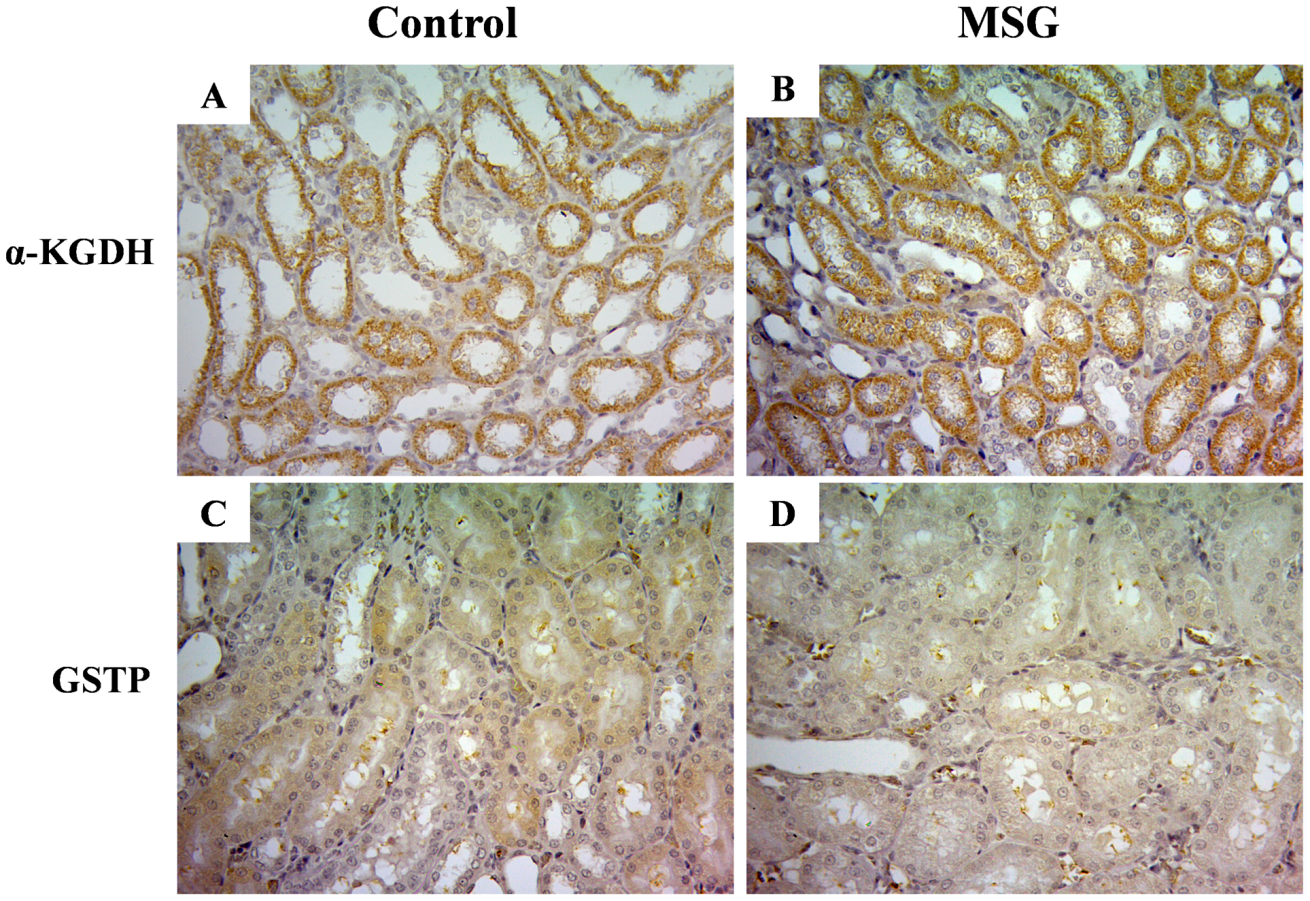
Immunohistochemistry staining of α-ketoglutarate dehydrogenase (α-KGDH) in kidney tissues of control (A) and MSG treated (B) groups and of glutathione S-transferase P (GSTP) in kidney tissues of control (C) and MSG- treated (D) groups. The images shown are representative of three independent experiments.

**Figure 3:**
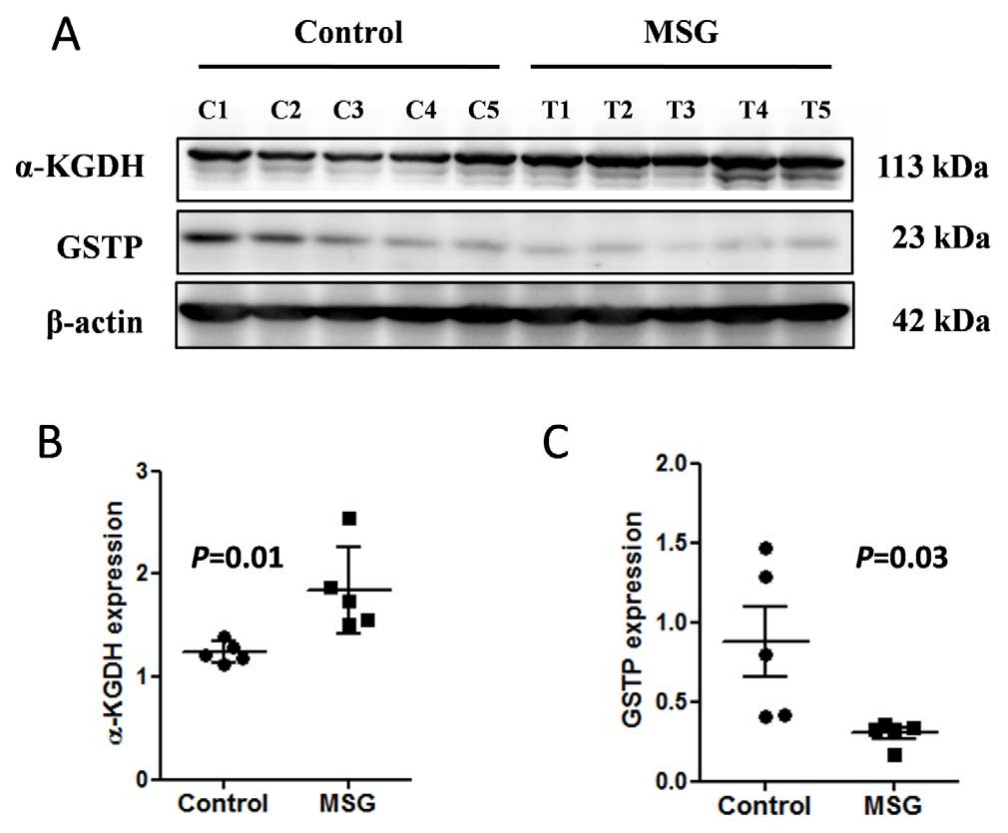
Western blot analysis of α-ketoglutarate dehydrogenase (α-KGDH) and glutathione S-transferase P (GSTP) in the kidney tissues of control and MSG treated groups (A), Quantitative analysis indicated that α-KGDH expression was significantly higher in the kidney of the MSG-treated group than that of control (B), whereas, GSTP expression was found significantly lower than that of control (C). The blots shown are representative of two independent experiments. C1–C5 and T1–T5 indicate animals in the control and MSG-treated groups, respectively.

Among 8 spots, 5 were up-regulated and 3 were down-regulated (**Table 1**). The identified proteins belonged to three functional categories: (a) a cellular enzyme involved in cell redox homeostasis, glutathione S-transferase P (GSTP), was down regulated, (b) a protein related to the stress response, heat shock cognate 71 kDa protein, was up-regulated, and (c) the metabolism associated enzymes phosphoserine phosphatase and phosphoglycerate kinase were down-regulated, whereas cytosolic glycerol-3-phosphate dehydrogenase, 2-amino-3-carboxymuconate-6-semialdehyde decarboxylase, α-ketoglutarate dehydrogenase (α-KGDH) and succinyl-CoA ligase were up-regulated. Immunohistochemistry staining corroborated the proteomic data showing higher expression of α-KGDH **(** **Fig. 2A, 2B** **)** and lower expression of GSTP **(** **Fig. 2C, 2D** **)** in MSG-treated kidney compared to the controls. We confirmed by the Western blot analyses that α-KGDH **(** **Fig. 3A, 3B** **)** and GSTP **(** **Fig. 3A, 3C** **)** were up-and down-regulated, respectively, in the kidney of MSG-treated rats compared to controls.

**Table 1:** List of renal proteins with significantly altered expression after long term chronic MSG treatment.

Spot no.	Protein	Accession No.	Theoretical PI/M_w_ (kDa)	MASCOT Score	MSG/control ratio
24	Glutathione S transferase P	gi|25453420	6.89/23.652	414	1.5↓
36	Phosphoserine phosphatase	gi|57527332	5.49/25.180	239	2.4↓
74	2-amino-3-carboxymuconate-6-semialdehyde dehydrogenase	gi|19705473	6.03/38.465	314	1.7↑
176	α-ketoglutarate dehydrogenase	gi|62945278	6.3/117.419	1224	1.2↑
208	Succinyl-CoA ligase	gi|51260799	7.57/47.042	960	1.2↑
211	Heat shock cognate 71 kDa protein	gi|13242237	5.37/71.021	1287	1.8↑
227	Glycerol-3-phosphate dehydrogenase	gi|57527919	6.16/38.112	421	1.3↑
242	Phosphoglycerate kinase	gi|206113	7.59/44.925	1055	1.7↓

## Discussion

To understand the regulatory mechanisms governing key proteins involved in the chronic MSG-induced renal toxicity, a gel-based proteomic approach was applied to examine changes in the renal proteome. Although a series of proteins in the kidney are dysregulated under various pathophysiological conditions, there is little information about the alteration of renal protein expression after chronic over-consumption of MSG. 2-D gel electrophoresis have the limitation of not being able to detect some low abundance proteins, basic proteins or insoluble membrane associated proteins [10]. Nevertheless, the present results show alterations of the renal proteome after chronic MSG intake.

We found eight proteins whose expression changed significantly in the kidney of MSG-treated rats compared to controls, three of which are involved in oxidative stress. Several extracellular and intracellular factors including nutrient metabolism affect production of oxidative stress. However, redox homeostasis is maintained by an antioxidant defense mechanism involving several enzymes such as glutathione S-transferases (GST). These are family members of the phase II detoxification enzymes involved in the conjugation of reduced glutathione to a highly diverse group of compounds. The products of GST catalysis are more water-soluble promoting ROS detoxification and thereby protecting tissues from oxidative damage [11]. In mammals, GSTs are divided into eight families [12]. Given that MSG enhances oxidative stress, one might predict that GST would be induced in order to increase the capacity to restore redox homeostasis. However, we found in this study that GSTP was significantly decreased in the MSG-treated rat kidneys compared to the control. The exact mechanism by which chronic MSG intake causes the decrease of GSTP level in the kidney is unclear. Supporting our findings, it has been reported that GSTP transcript level was markedly down-regulated in a mouse model of asthma following oxidative stress by allergen challenge [13]. Furthermore, the decrease in GSTP expression was associated with a decrease in the total GST activity in the lungs of mice [13] similar to what Paul *et* *al*. observed in the kidney tissue of MSG treated rats [2].

In the present study, the most remarkably changed proteins were the metabolism-related enzymes. Phosphoserine phosphatase (PSP) was significantly down-regulated in the kidney of MSG treated rats. This cytoplasmic enzyme splits o-phosphoserine to serine and phosphate [14]. Serine can be further metabolized to yield glycine, cysteine and pyruvate [15]. Glycine and cysteine are precursors for glutathione synthesis. Furthermore, in this study the down-regulation of PSP may be the defense mechanism of kidney cells. L-glycine and D-serine are the co-agonist at the *N*-methyl-*D*-aspartate (NMDA) subtype of the iono-tropic glutamate receptor [16]. Since kidney tissue expresses NMDA receptors [17], it could be a mechanism to restrict the activation of glutamate receptor by decreasing co-activators, namely glycine and serine.

The up-regulation of cytosolic glycerol-3-phosphate dehydrogenase (G3PD) seen in the kidney of MSG-treated rats suggests the activation of a shuttle system by MSG. G3PD plays a role in glycerophosphate shuttle that conveys cytosolic NADH into mitochondria for oxidation. Cytosolic G3PD transfers reducing equivalents from NADH to dihydroxyacetone phosphate, producing glycerol-3-phosphate (G-3-P). G-3-P is a key metabolite connecting glycolysis, lipogenesis and oxidative phosphorylation, suggesting MSG alters the energy of metabolism [18]. Since phosphoglycerate kinase (PGK) is an important enzyme in the glycolytic pathway, down-regulation of PGK found in MSG-treated group may suggest that MSG slows down glucose catabolism. PGK plays an important role in glycolysis, producing ATP at the substrate level of phosphorylation. Regulation of this protein is thereby controlled by ATP or energy levels within the cell. This may be supplied by amino acid catabolism, as evidenced by the up-regulation of 2-amino-3-carboxymuconate-6-semialdehyde decarboxylase (ACMSD), an enzyme in tryptophan catabolism in MSG-treated rats. ACMSD is detectable in the liver and kidney, however its activity is much higher in the latter [19], [20]. ACMSD is known to be up-regulated in rats fed a high protein diet [21] and in this study, both control and MSG treated rats were fed a protein-rich diet (24%). MSG may increase the proportion of glutamate in the amino acid pool, leading to increased amino acid catabolism [22].

**Figure 4:**
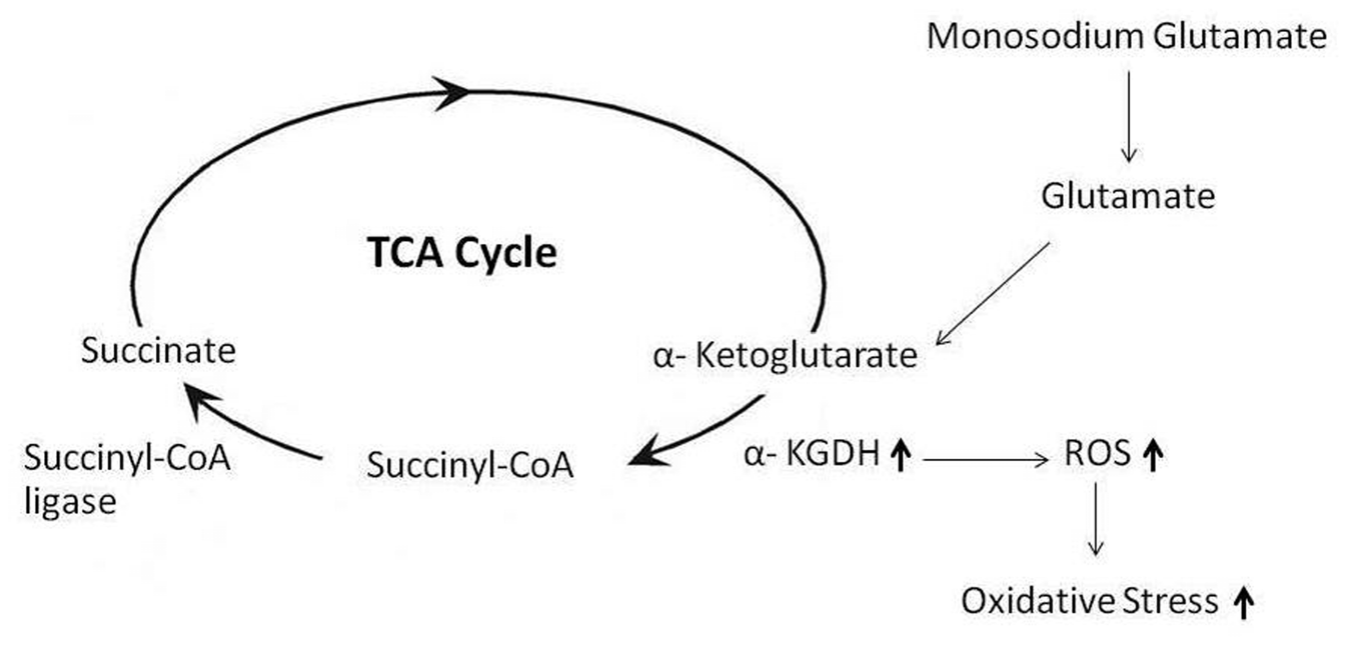
Proposed model of MSG-induced ROS production in rat kidney. The higher level of glutamate on chronic MSG intake accelerates TCA cycle. The increased level of α-KGDH may stimulate ROS production hence oxidative stress occurs in the kidney of the MSG-treated rats.

The significant increase of the expression of two enzymes, α-ketoglutarate dehydrogenase (KGDH) and succinyl-CoA ligase (Suclg2), suggested that continuous administration of MSG accelerates the tricarboxylic acid (TCA) cycle. We speculated that this increase was caused by accelerated amino acid catabolism especially glutamate. It is well-recognized that α-ketoglutarate dehydrogenase plays vital roles in multiple pathways of energy metabolism and biosynthesis. The enzyme α-KGDH is also able to generate ROS [23], [24], [25]. We hypothesize that the increase of α-KGDH in MSG-treated rats may relate to glutamate-stimulated ROS production (**Fig. 4**) that has been previously reported in brain tissue [26]. Succinate, a product of a Suclg2 catalyzed reaction, can result in high rates of H_2_O_2_ production. Increased level of Suclg2 and oxidative stress has also been reported in rat kidney exposed to perfluorododecanoic acid [9].

Oxidative stress plays a key role in the pathophysiology of a variety of clinical and experimental progressive renal diseases [27]. In a previous study, the levels of antioxidant enzymes were decreased and, conversely, a lipid peroxidation marker was increased in MSG treated rat kidneys, suggesting an increase in oxidative stress [2]. Here, we show heat shock cognate 71 kDa protein (Hspa8 or HSC 70) was up-regulated in the kidneys of MSG-treated rats, also suggesting an increase in oxidative stress. Involvement of HSC 70 strengthens the argument that oxidative stress is the main culprit behind the MSG toxicity in chronic MSG intake. It is possible that glutamate contributes fuel to the TCA cycle and modulates the redox state of the cell. High glutamate concentration may increase the mitochondrial proton gradient as a result of over production of electron donor by the TCA cycle, which may in turn increase production of mitochondrial superoxide [21].

One limitation of the current study is that we did not measure serum glutamate levels. However, we previously reported altered kidney function in MSG-treated rats for 9 months compared to controls, including significantly increased renal fibrosis [6]. Further experiments are needed to address the limitations of the current study.

In conclusion, the present results can contribute to clarify the mechanisms underlying MSG toxicity and alteration in renal metabolism, by indicating key proteins that should be better addressed in future studies.
